# Exploring sentiments and topics in Extended Reality learning environments: A comparative study

**DOI:** 10.1371/journal.pone.0327311

**Published:** 2025-07-14

**Authors:** Hosam Al-Samarraie, Samer Muthana Sarsam, Ahmed Ibrahim Alzahrani, Hanan Aldowah

**Affiliations:** 1 School of Design, University of Leeds, Leeds, United Kingdom; 2 School of Management, Coventry University, Coventry, United Kingdom; 3 Computer Science Department, Community College, King Saud University, Riyadh, Saudi Arabia; 4 School of Management, Universiti Sains Malaysia, Penang, Malaysia; De la Salle University, PHILIPPINES

## Abstract

This study explored online users’ sentiments about the use and application of Extended Reality (XR) in higher education. X social media platform was used as the main source for assessing users’ perceptions of XR in teaching and learning. A topic modelling approach was used to identify and compare main themes and topics in relation to the use of Virtual Reality (VR) and Augmented Reality (AR)/Mixed Reality (MR). The sentiment and polarity of each topic were assessed and compared across these technologies. The results revealed three themes for VR (self-directed learning, creativity-promoting environments, and challenges and concerns) and three themes for AR/MR (guided and oriented learning experience, playful and flexible learning experience, and challenges and concerns). The results also demonstrated significant differences in users’ sentiments, with VR use in higher education achieving higher positivity, anticipation, trust, and joy compared to AR/MR. Findings from this study are unique in the sense that they offer a comparative perspective on XR inclusion in university teaching and learning. The findings can direct policy makers in higher education about the suitability and feasibility of using certain XR technologies in facilitating their digital transformation goals.

## 1. Introduction

The recent advancements in Extended Reality (XR) have opened new means for universities to experiment and plan various learning activities. Higher education institutions have promptly designed and developed pilot projects [1,2] to understand the impact of XR in certain teaching and learning settings. The literature search revealed few large-scale projects conducted to map learners’ and educators’ perceptions of XR in a university context. For example, Kluge, Maltby [3] conducted a study to examine aspects related to users’ acceptability, value areas, barriers, and opportunities for the adoption of XR in teaching. The authors employed an online survey and targeted interviews to determine users’ readiness for broad adoption of XR technologies in university education. Kluge et al. concluded a slow integration of XR applications into the curriculum. Another study by Tunur, Hauze [4] explored students’ perception of using XR-immersive labs. The authors reported that XR-immersive labs were found to offer students a unique aspect of learning through increased perceptual arousal or inquiry arousal, as well as confidence and overall satisfaction. However, the conclusions drawn from these pilot studies were limited in focus and scope, raising questions about their generalizability to the wider spectrum of higher education. In addition, a number of reviews have been published about the potential of VR, Mixed Reality (MR) in higher education. Most of these reviews [5–10] have addressed the potential of VR/MR in shaping students’ learning experience. They also reported some key challenges in relation to the technical capability and immersive readiness of educational institutions. Despite previous reviews on this topic, there remains a lack of understanding of users’ sentiments and emotions regarding the use of XR technologies, which are known to be important factors in assessing users’ adoption of technology.

Therefore, this study seeks to offer a generic perspective about the role of XR in higher education. To achieve this, we opted to use topic modelling and sentiment analysis to identify and map main themes, topics, and emotions of online users on this topic. We used sentiment analysis and topic modelling approaches to answer two key questions: 1) What are the themes and topics associated with the use of XR in higher education? and 2) What are the sentiments of Twitter users toward XR in higher education? Outcomes from this work can provide a deeper understanding of users’ sentiments/emotions and polarity toward the technology in a university setting. The study also sought to offer comparative insights for educational policymakers to consider when approaching VR/AR/MR integration into future curriculum development. This paper is organised as follows: Section 1 introduces the work and main research questions; Section 2 reviews the literature on opinion mining in higher education; Section 3 discusses the method used in this study (e.g., data collection, filtering, topic modelling, and sentiment analysis); Section 4 presents the results of topic modelling and sentiment analysis; Section 5 discusses the practical implications of the work; Section 6 outlines the limitations and directions for future research; and Section 7 concludes the paper.

## 2. Opinion mining in higher education

Scholars consistently emphasize the effectiveness of analysing feedback collected from students through diverse channels, including questionnaires, surveys, social media, and forum posts. These approaches offer valuable insights into users’ perceptions, yet they also have their limitations. While structured feedback, such as pre-planned surveys conducted at the department or university level, is available, a substantial portion of individuals’ opinions is conveyed through free-text comments on social media networks. However, the vast volume of online comments makes manually processing them a time-consuming and impractical approach.

Users’ posts on social media sites can provide valuable insights into their emotions and feelings [11], which is crucial for making informed decisions in the future. However, it is essential to facilitate the automatic processing and analysis of data. This automated processing of social media posts/comments is commonly referred to as opinion or sentiment mining (or analysis) [12].

In general, individuals express emotions differently based on various psychological, social, and personal factors. This is why different machine learning tools have been extensively utilised to detect emotions from text. Several studies [13,14] have explored the use of emotion profiles from text or images to characterise events and situations. On Twitter, researchers have widely analysed users’ emotions and sentiments to gain valuable insights into their perceptions and feelings. This is because sentiment analysis helps identify subjective information, revealing an individual’s attitude towards specific topics or experiences. On the other hand, emotion analysis delves into contextual emotions, categorising them into predefined emotion classes. To train machine learning algorithms in recognising sentiments, we employed a topic modelling approach to extract and identify key themes and topics associated with the use of VR and AR, including MR. The specific method utilised for this purpose is explained in the following section.

## 3. Method

**Fig 1:**
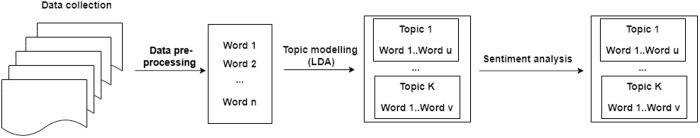
Research procedure.

We used a systematic approach encompassing four primary steps: data collection, data pre-processing, topic extraction and modelling, and sentiment analysis (see Fig 1). Throughout this study, all these steps were executed using RStudio, a software environment that seamlessly integrates with R, a programming language tailored for statistical computing and graphics. We adhered to X’s scraping policies and collected data via X’s Academic API. The data collection methods comply with these policies and align with ethical research practices. As race, ethnicity, and gender are difficult to identify and retrieve directly from X, sentiment biases were not considered in this study. This study uses masked API data generated from social media sites for which ethical approval is not required.

### 3.1 Data collection

Using the Twitter basic streaming Application Programming Interface (API), we successfully extracted 126,479 English-language tweets posted between January 1, 2020, and April 30, 2023, a period marked by the rise of XR applications in higher education. The collection of tweets was accomplished by employing a combination of keywords, including “extended reality,” “XR,” “AR,” “augmented reality,” “VR,” “virtual reality,” “mixed reality,” “MR,” and “immersive,” in addition to “higher education,” “university,” “undergraduate,” and “postgraduate,” ensuring their relevance to our research objectives. It is worth noting that we adhered to the standard Twitter research ethical guidelines while gathering the data. Our initial inspection of the retrieved tweets showed several irrelevant comments that mentioned ‘XR’ and ‘university’ or ‘universities’ in contexts unrelated to learning or teaching, such as marketing, customer service, and satisfaction surveys. These tweets discussed the use of AR/MR and VR in general service satisfaction, bearing little or no relevance to the higher education context. To address this issue, we carefully excluded a selection of terms (e.g., -service, -business, -faculty, -sport, -job, -methods, -finance, -technique) that had led to non-meaningful tweets. After making these adjustments to the search criteria (e.g., specifying the discipline or teaching practices), we retrieved 121,750 tweets. A final manual inspection of 500 tweets revealed a relevant mix of views and opinions more closely aligned with XR in higher education.

### 3.2 Data pre-processing

This study performed screening on all the collected tweets using various filtering approaches. For instance, we excluded retweets (n: 88,027) from the initial data collection, which led us to 33,723 tweets. We also employed tokenisation to facilitate the extraction process of relevant words from the collected tweets, achieved by dividing sentences into individual words. The extracted words were then utilised to create a dictionary, forming the basis of our main corpus. To attribute significance to each word, a weighting scheme known as term frequency–inverse document frequency (TF-IDF) was employed. The calculation of the TF-IDF weight was performed according to the method outlined by Bhattacharjee et al. (2019). Following this process, we achieved a collection of words related to the tweets (features).

To further refine the data, we decided to eliminate symbols (@), URLs, and hashtags, retaining only the essential tweet content to ensure high consistency (see Table 1 for the detailed breakdown). This includes the elimination of special characters (e.g.,!%$#& *?,/.;”) using regular expression techniques, and the removal of non-essential words through the use of the stopwords list technique (a predefined set of words that commonly appear but are irrelevant to the analysis). Following these steps, we divided the data into VR (n:19,401) or AR/MR (n: 7,893) tweets.

**Table 1:** Characteristics of the corpus of tweets.

Variables	Value/Statistic
Total related tweets	121,750
Period	Jan 2020 – Apr 2023
Removed Hashtags (#) no.	3431
Removed Mentions (@) no.	2998
Removed Retweets no.	88,027
Width (characters) before the data pre-processing stage	• min = 10 • average = 275 • max = 280
Width (characters) after the data pre-processing stage	• Min = 9 • Average = 130 • Max = 280
Favourites count	• min = 0 • average = 4.29 • max = 1450
Total clean/usable tweets	27,294 • VR = 19,401 • AR/MR = 7,893

### 3.3 Topic modelling

The primary XR-related topics in higher education were identified using the Latent Dirichlet Allocation (LDA) algorithm, following the recommendation of Ostrowski [15]. LDA is an unsupervised method that treats each document as a combination of topics, producing topic summaries with a discrete probability distribution over words per topic and estimating distinct distributions of topics per document [16]. The use of topic modelling based on LDA was primarily due to its capacity to unveil latent XR classification systems, which may or may not overlap with established XR classifications. In addition, the underlying LDA model operates on the probabilistic generation of textual information, which allowed us to represent XR topics through distributions over words, and conversely, words through distributions over topics. In the context of XR tweets, each tweet is allocated a set of topics, and the words within the tweet are subsequently assigned to these identified topics. Therefore, specific XR usage words are more likely to appear in particular learning situations/conditions compared to others.

The number of topics generated by the LDA was identified using the elbow method [17,18]. The performance of the LDA was evaluated using the perplexity measure [19,20]. Perplexity is considered one of the most popular measures in language modelling for estimating the predictive accuracy of observed texts, such as tweets in our case. To ensure the accuracy of the output, we followed two steps: 1) evaluating the coherence and exclusivity of each topic, and 2) manually inspecting the model’s output for interpretability. During this process, the first and second authors independently provided their opinions on each topic by reviewing topic-related tweets. We then assigned appropriate labels (see section 4.1) to the identified group of topics generated by the LDA algorithm for either VR or AR/MR. We employed probabilistic inference from topic modelling to uncover the underlying labels in the text and interpret the topics. The probability of a given topic was determined by the proportion of terms attributed to that topic across the entire corpus. We then used a measure of topic coherence and exclusivity to select and retain the best model.

To ensure the validity of our labelling, we utilised the kappa statistic method to evaluate the level of agreement (e.g., agree and disagree) between two external evaluators in the field of digital innovation and higher education. The evaluators were given 300 randomly selected tweets to identify suitability of topics to each category (e.g., AR/VR), and the validation results revealed a 91% agreement among the evaluators, indicating a high level of consensus.

### 3.4 Sentiment analysis

At this stage, users’ sentiments were extracted from their tweets using two main techniques: the “NRC Affect Intensity Lexicon” and “SentiStrength”, implemented via the Waikato Environment for Knowledge Analysis (WEKA) software. The NRC Emotion Intensity Lexicon method was selected for this study due to its comprehensive coverage of a wide range of emotions expressed in tweets, as opposed to a binary sentiment lexicon. This lexicon comprises a list of English words along with their associations with various emotions, including disgust, surprise, sadness, anger, fear, joy, anticipation, and trust [21]. These emotions were deemed relevant to the focus of this study, as they represent the broad spectrum of emotions that an online learner may experience when using XR technologies. According to many previous studies [22,23], these emotions can elicit distinct cognitive and affective responses, which may influence individuals’ perceptions and use of XR learning environments. Other types of emotions (e.g., excitement and curiosity) were not directly investigated in this study, primarily due to the limitations of the NRC lexicon.

We also assessed the polarity of the processed tweets using the “SentiStrength” technique, which applies a set of rules to combine the sentiment of individual words and determine the overall sentiment of the entire text. Specifically, we used SentiStrength to identify two types of tweet polarity: positive and negative. Sentiment is represented by numerical values ranging from −1 (not negative) to −5 (extremely negative) for negative sentiments, and 1 (not positive) to 5 (extremely positive) for positive sentiment [24].

To further validate the results of the NRC Affect Intensity Lexicon and SentiStrength, we followed the approach recommended by Biswas, Karabulut [25]. We compared the identified emotion and polarity labels with those generated by a more context-aware sentiment classifier, such as BERT (Bidirectional Encoder Representations from Transformers). BERT leverages transfer learning, where a pre-trained model is fine-tuned on task-specific labelled datasets, such as GoEmotions, to enhance sentiment analysis across various contexts. Although originally trained on a broad dataset, BERT’s ability to generalise allows for effective sentiment classification in our domain-specific dataset, enabling us to classify tweets into distinct emotion and polarity categories. In addition, to assess the alignment between our methods (NRC Affect Intensity Lexicon and SentiStrength) and BERT, we used Cohen’s Kappa. The results showed a Kappa value of 0.95 for NRC and 0.92 for SentiStrength, indicating a high level of agreement. This validation reinforces the reliability of NRC and SentiStrength for sentiment analysis in our study.

## 4. Results

To address participants’ demographics, we followed a two-step approach to infer whether the users were students. First, we examined the profile information provided through the Twitter API, assuming that students would often mention their university, course of study, or explicitly refer to themselves as “students” in their bio. Second, we analysed the tweet content itself, assuming that references to university life, exams, coursework, or campus events could indicate that the user was a student. Table 1 presents a summary of the characteristics of the corpus of tweets. From the table it can be seen that the total number of tweets was 27,294 (19,401 tweets on VR and 7,893 tweets on AR/MR). We removed 3,431 Hashtags (#), 2,998 Mentions (@), and 88,027 retweets from the dataset. The width (characters) before and after the data pre-processing stage is also presented in the table with an average Favourites count (the number of times a tweet has been marked as a favourite (or “liked”) by other users) of 4.29.

**Fig 2:**
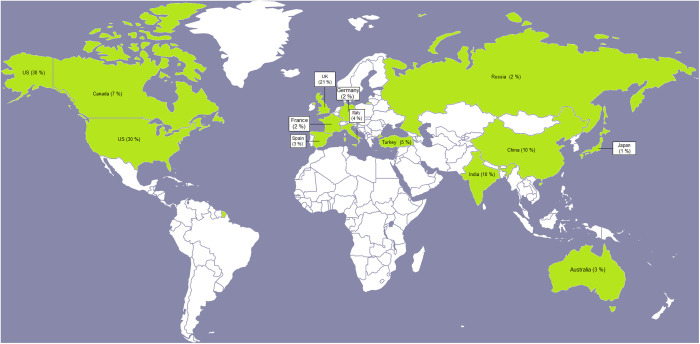
Distribution of the tweets across countries.

Fig 2 illustrates the distribution of tweets related to the application of XR in higher education across countries. The figure highlights a notable concentration of topics among users from the United States (US), United Kingdom (UK), India, China, and Canada. This trend may be attributed to technological advancements and dedicated efforts in these countries to establish effective pedagogy for metaverse learning. It is also important to note that this study did not consider the role of gender in XR use in higher education, primarily due to the complexities associated with identifying gender identity within a large dataset.

**Fig 3:**
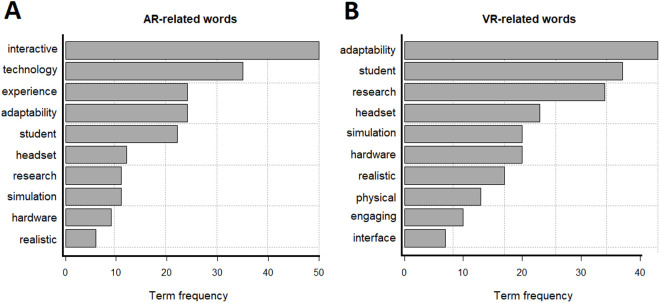
Most frequent words (> 4) observed in the corpus of tweets.

The most frequent words related to the use of VR and AR in higher education are illustrated in Fig 3. From the figure, it can be observed that users’ discussions and stories about VR in higher education primarily revolved around its adaptability and control. These critical benefits are believed to enhance users’ sense of control and realism. Interestingly, previous studies have extensively debated the suitability of VR in facilitating students’ learning compared to AR. This may be attributed to the limited data and conceptual focus of those studies, making it challenging to explore the multidimensional facets of VR utilization as a potential driver of a future educational revolution. In addition, the results showed that both engagement and the interface of VR were the least concerning or popular aspects in higher education. From an AR perspective, the most frequent words were pointing to the role of the technology in adding flexibility and facilitating interactivity between the learner and learning experience. This is reasonable giving the specification of AR technology in allowing users to directly interact and immersive with the learning content.

### 4.1 Topic modelling results

**Fig 4:**
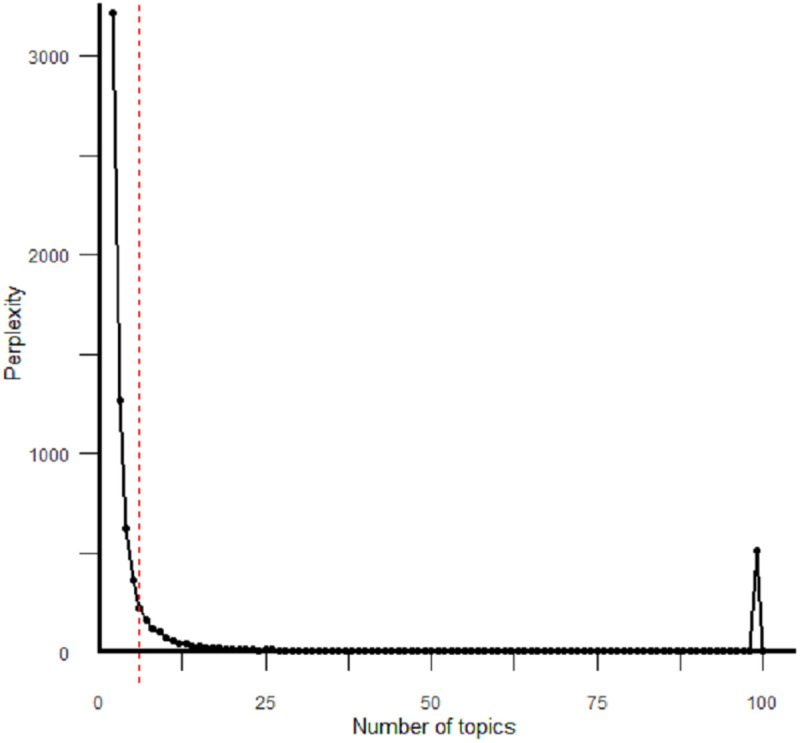
LDA performance over a range of topics (2 to 100) using the perplexity measure. The dotted line represents the selected cut-off (6 topics using the elbow method).

The perplexity results, as shown in Fig 4, indicated that the optimal cut-off point settled on three topics for VR and three topics for AR (including MR) (see section 4.3). The elbow method was used to estimate the optimal number of topics for this study, occurring at the point where further increases in the number of topics yield diminishing returns. The dotted line in the figure marks this point, indicating that beyond it, additional topics introduce complexity without meaningful improvements. Based on this analysis, six topics were selected as the optimal number.

Table 2 presents a summary of these topics along with their probability values and top 10 keywords. The ‘Mean Topic Probability’ was calculated using the ‘topicmodels’ package in R. This package generates the document-topic distribution as part of the LDA model output. The mean probability for each topic was derived directly from this distribution, which provided an overall measure of how dominant each topic was within the dataset.

**Table 2:** Summary of topic modelling.

XR technology	Mean topic probability	Top 10 related words	Themes
VR	0.74	*Interactive, self-assessment, engaging, effective interaction, hands-on, realistic, ownership, reflection, personalized, adaptability*	Self-directed learning
VR	0.76	*immersive, imagination, solutions, changing, interesting, augment, thinking, deep, curiosity, exploration*	Creativity-promoting environment
VR	0.88	*discover, learner, research, training, time, sickness, cognition, attention, haptic, cost*	Challenges and concerns
AR	0.72	*guided, amazing, engaging, fun, adaptability, improving, follow, adjust, find, clear*	Guided and oriented learning
AR	0.81	*exciting, fun, adaptability, game, continuity, explore, joy, control, entertaining, creativity*	Playful and flexible learning
AR	0.77	*realism, interactivity, practice, physical, engaging, transforming, understanding, compare, ambiguity, learning contents*	Challenges and concerns

#### 4.1.1 VR-related topics.

We found three main themes associated with VR use in higher education: self-directed learning (mean: 0.74), creativity-promoting environments (mean: 0.76), and challenges and concerns (mean: 0.88).

a. *Promoting self-directed learning*

The results revealed three distinctive topics associated with this theme. The first topic (mean: 0.73) discussed the effectiveness of VR learning environments in enhancing learners’ interactivity and engagement with complex tasks. For instance, 42% of users highlighted how the VR experience helped them navigate complex learning materials that would not have been possible in a real-world context. VR was also reported to facilitate knowledge integration and skills development among learners with specific needs. Some of the stories related to this topic addressed the benefits of VR in helping learners build coping strategies when dealing with sophisticated concepts. This finding aligns with previous debates, such as the one presented by Chang and Hwang [26], who argued that VR could provide trainees with opportunities to experience problem-solving within the context of interactive learning. It’s worth noting that most studies reporting evidence of VR’s role in promoting coping strategies have been largely conducted in training and health contexts [27]. This raises questions about the extent to which VR environments can support learners in developing solutions to learning challenges within a higher education context.

The second topic (mean: 0.66) centred on the realism and reflective capabilities of VR. This was evident in users’ tweets where they expressed interest in using VR to visualize various phenomena, ontologies, anatomical structures, data, and theoretical problems. Approximately 34% of users reported that incorporating VR into university contexts, especially in fields like medicine and engineering, allowed them to enhance their awareness and self-reflection regarding learning processes that require lifelike visualization. While previous studies have extensively discussed VR realism and the development of self-reflection capabilities from various perspectives in different contexts [28,29], there is limited research that specifically explores the relationship between the sense of VR realism and the development of self-reflection capabilities within a university setting. This is an area that warrants further investigation, particularly in the fields of medicine and engineering.

The third topic (mean: 0.60) discussed the adaptability of the learning experience to users’ needs through machine learning and pedagogical scenarios. Some of the retrieved tweets (9%) related to this topic highlighted the potential of VR in developing pedagogical actions that can support learners with specific learning styles. The adaptability of the learning experience can empower learners to perceive ownership, actively engage with the learning task, and perceive it as tailored to their individual needs. From these discussions, it can be concluded that VR holds significant promise for fostering students’ self-directed learning. Furthermore, there is still a dearth of research on how the aforementioned topics can be integrated into the design process of future adaptive VR experiences.

b. *Creativity-promoting environment*

A total of two topics were mainly discussed in relation to this theme. The first topic (mean: 0.69) looked into the role of VR in stimulating sense of imagination and curiosity, especially in Art subjects. Some users (26%) noted that using VR introduced a sense of positive challenge to the learning process, which led learners to identify and generate new approaches to the learning topics. This can also be attributed to the flexibility of VR environments, which facilitate users’ navigation across various spaces, thereby fostering a greater sense of freedom for learners to express and explore new ideas. Other users (14%) found that VR enabled them to express themselves through avatars, which wasn’t possible in real-world settings. Another aspect highlighted in tweets (7%) was the collaborative nature of VR spaces, where users could share and compare ideas related to specific themes.

The second topic (mean: 0.64) explored the potential of VR in encouraging students to seek innovative solutions to specific problems. For example, upon reviewing several tweets (16%), it was evident that students use VR to analyse a range of problems and solutions from multiple perspectives within collaborative spaces. Furthermore, our findings indicated that some tweets (10%) discussed the flexibility of AI-enabled VR environments in enabling learners to experiment with various scenarios and identify the root causes of certain learning challenges. This form of intervention is believed to enhance learners’ ability to synthesize provided information and refine their judgment.

The outcomes from this theme are believed to contribute to previous efforts [30–34] aimed at understanding how specifically designed VR experiences can stimulate users’ creativity. It illustrates how certain VR modalities, such as immersion and interactivity, can provide the means to inspire learners’ imagination and exploration of solutions.

c. *Challenges and concerns of VR in higher education*

Despite the wide range of opportunities offered by VR, several tweets highlighted various challenges and concerns that policymakers, educators, and practitioners may need to consider when adopting this technology in education.

The first topic (mean: 0.78) discussed motion sickness issues, which some users posted or shared across their networks as a drawback of using VR in learning. Our results indicated that 36% of tweets on this topic discussed time, orientation, and headset comfort as the main predictors of motion sickness in VR. This issue has been extensively discussed in previous studies as a setback in promoting the technology in education. Another stream of tweets (22%) reported that individual learning might trigger motion sickness when attempting to concentrate on a task for a longer period of time. This finding is rarely explored in previously published work on VR motion sickness. Some suggestions found in the replies to tweets about this topic indicated the potential of tactile stimulation to create a homogeneous cognitive state among learners spending long periods learning or collaborating in VR spaces.

The second topic (mean: 0.70) discussed issues related to students experiencing split attention, visual fatigue, and burnout when learning with VR. 32% of the tweets mentioned that VR environments that incorporate a wide range of activities and navigational needs can result in learners experiencing split attention. It’s worth mentioning that there is limited research on the split-attention effect in VR, and the input from tweets did not provide us with a deeper understanding of the measures needed to reduce it. However, our understanding suggests that the design characteristics of VR environments might largely contribute to this experience. In addition, 25% of tweets revealed that students are likely to experience visual fatigue when they are expected to concentrate on moving learning objects or shift from one presentation style to another. The tweets indicated the potential for burnout among students when they are asked to use the technology for extended periods. The specific duration or timing in relation to this use was not clear or expressed in users’ tweets, making it difficult to gain a deeper understanding of this aspect. Findings from this topic offer new insights into the impact of using VR on users’ cognitive state from different perspectives.

The third topic touched on affordability and training concerns when considering VR technology in a university learning. Precisely, 31% of the tweets discussed cost-associated obligations when deciding to engage in a VR experience. Some of the tweets were mapped around the limited fund and training resources a higher education institution put to support the integration of VR in teaching and learning. Another stream of tweets (14%) looked into the lack of initiatives and political will to maximize the use of VR technology beyond providing a short demonstration of specific concepts or topics. Other tweets indicated the need for targeted funding to enhance the efficiency of VR technology in disciplines that require special learning conditions and hands-on experience to develop critical skills. This perspective has been widely shared in settings related to medicine and science subjects [35]. These findings offer important directions for policy makers in higher education to consider when mapping the integration of VR in their digital transformation agenda.

#### 4.1.2 AR-related topics.

Tweets on AR were also retrieved and categorised to provide a holistic understanding of the topics associated with its use in higher education. Three themes were associated with AR use in higher education: guided and oriented learning experience (mean 0.72); playful and flexible learning experience (mean: 0.81); and challenges and concerns (mean:0.77).

a. *Guided and oriented learning experience*

The majority of tweets (47%) expressed the effectiveness of AR applications in enabling learners to observe and engage with the learning process through mobile devices and MR headsets, such as HoloLens. Some tweets highlighted the effectiveness of MR in providing self-guided learning through structured interactions with a series of learning modules. The use of AI to help learners orient themselves and identify and reflect on learning objects in an immersive environment has been shown to guide learners in discovering and refining their understanding of engineering concepts (e.g., logic building and advanced foundation of engineering). For instance, 10% of tweets emphasize the feasibility of AR in promoting learners’ sustained engagement with tasks, which, in turn, can contribute to their comprehension of the characteristics and features of learning objects. Our analysis of user tweets suggests that the relatively low complexity of operating and interacting with 3D objects in an AR environment may explain learners’ preference for this technology when studying complex concepts. However, this preference seems to be specific to certain disciplines and may not be generalizable to others. Another group of tweets (16%) revealed that AR-based tactile sensations, primarily in an MR setting, can enable vocational learners to immerse themselves deeply in the learning process through the sense of touch, often delivered through vibration and pressure. Some users (7%) reported that this haptic-augmented experience can greatly motivate and excite users to explore complex topics that demand a deeper system-level understanding such as general science concepts.

From these findings, it can be concluded that the opportunities AR/MR offers to specific specialties would enable learners to align their learning experiences more closely with their individual needs. This is supported by previous studies in medical education, such as Dhar, Rocks [36], which addressed how AR-based teaching programs could enhance the experiences of medical students by improving their knowledge, understanding, practical skills, and social skills. Despite that, the implementation of AR/MR has been limited and not widely adopted across many disciplines. Some of the reasons for this are discussed in Section c.

b. *Playful and flexible learning experience*

Our analysis of tweets related to this topic revealed a positive sentiment towards the use of AR in creating an engaging learning environment by incorporating game-based activities into the learning process, particularly in subjects like programming and language. The results showed that 24% of the tweets highlighted the potential of AR in enhancing learners’ sense of presence and control during the learning activities. This trend was observed across various disciplines, including tourism, health, engineering, language, library studies, arts, and sports, in contrast to VR use, which was found to be concentrated in specific areas. It is believed that the engaging nature of gamified learning experiences in AR allows learners to explore and enjoy the educational tasks without concerns about burnout or distractions, as can sometimes occur with VR. Other aspects related to the adaptability and attractiveness of AR were expressed by online users (17%). These means are believed to have shaped learners’ sense of the perceived flexibility of the technology in navigating their learning journey. Some tweets (5%) described the use of MR in stimulating students’ creativity when working on individual and collaborative projects. The flexibility offered by the induced sense of control is believed to have influenced learners’ enjoyment and creativity. This is in line with previous findings [37,38] on AR use in facilitating the state of flow among users due to its flexibility in integrating coherent strategies appropriate for maintaining the level of challenge among users. From the flow theory perspective, when the challenge level in a learning situation is within the skills and capability of the learner, he/she is likely to gain a higher sense of control and enjoyment [39,40]. It is also reasonable to assert that AR/MR might offer the flexibility for universities to transition into their immersive teaching and learning agenda by gradually introducing its application across disciplines. This requires a strategic action plan that would help higher education providers achieve their integration goals and objectives.

c. *Challenges and concerns of AR use in higher education*

A number of challenges related to the use of AR in a university context were identified from our search of users’ tweets. We found that although the technology has great potential to enhance students’ learning experiences in various disciplines, there is still much to be done for higher education institutions to increase its adoption and use by faculty members and students. For example, the majority of tweets (37%) revealed a lack of initiatives to standardize and promote the use of AR technologies in disciplines with multiple hands-on exercises. This was most notable in medicine and engineering subjects, where users highlighted the absence of integrated AR presentations in the curriculum design. This absence may reduce instructors’ willingness to incorporate the technology into their teaching practices.

On the other hand, 21% of tweets mentioned financial factors as a hindrance to facilitating AR integration into teaching and learning. This primarily involves providing the necessary training for staff and students, including the development of skills for digital education units to design AR experiences tailored to students’ specific needs. Another 17% of tweets discussed the difficulty of interacting with marker-based objects to navigate through the learning space, especially when the task requires learners to remain in one place. Another stream of tweets (5%) added that marker less solutions can still introduce some elements of ambiguity among learners regarding where objects will appear and how they can be tracked when the learning activity begins. The use of this technology can impose physical limitations on learners’ ability to maintain long-term engagement and concentration with the content, as it involves constantly changing their perspective to locate visual cues and content.

The results also revealed that 7% of users shared concerns about the feasibility of AR technologies in transforming current learning practices. Precisely, our analysis of the tweets revealed that some users were not convinced that the technology would allow them to develop their knowledge collaboratively. This can be attributed to the difficulty of coordinating or even establishing collaboration when multiple activities are taking place simultaneously in an AR environment. Meanwhile, the learning flow in an AR space is not yet capable of adjusting the learning experience according to users’ current emotional or cognitive needs. Although there have been some efforts in the past to use AI with AR [41], most of the focus has been on the detection and recognition of objects. The literature also indicates a notable lack of the use of AI to enhance learners’ understanding of complex tasks in AR [42]. With the slow integration of AI into AR, the technology’s use in higher education might not yet be well-positioned to support the diverse needs of learners in different disciplines.

**Fig 5:**
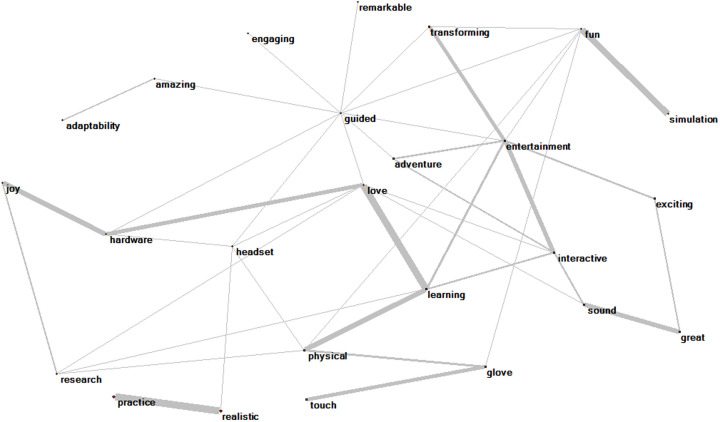
VR network model.

**Fig 6:**
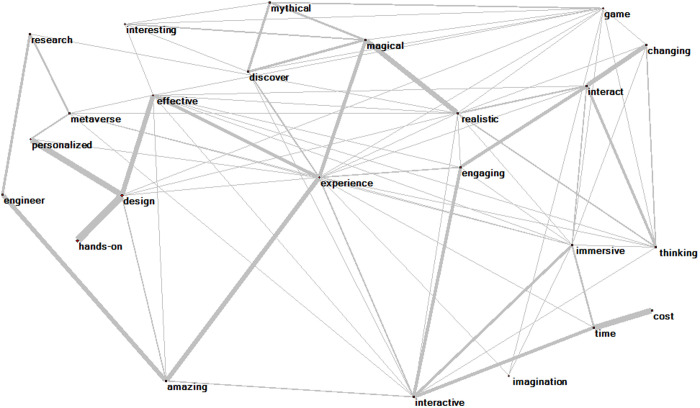
AR network model.

Fig 5 and Fig 6 provides a holistic illustration of keywords and associations in relation to the use of XR in higher education. The width of the connecting lines is proportional to the frequency of word co-occurrences, with higher density indicating more frequent word co-occurrences.

### 4.2 Sentiment analysis results

**Fig 7:**
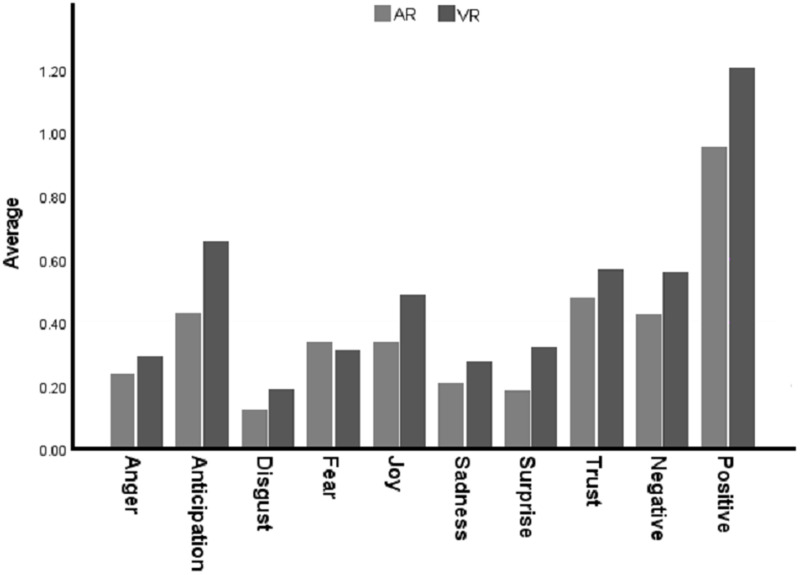
Sentiment within the corpus.

Our sentiment analysis results are summarized in Fig 7. From this figure, it can be observed that online users’ opinions regarding the use of VR in higher education were predominantly positive (M = 1.20, SD = 0.19), as well as for the use of AR (M = 0.95, SD = 0.16). The results also indicated higher emotional responses related to the use of VR, particularly in anticipation (M = 0.65, SD = 0.06) and trust (M = 0.57, SD = 0.08), compared to AR (anticipation: M = 0.43, SD = 0.09, trust: M = 0.48, SD = 0.13). Furthermore, the ANOVA test analysis revealed significant differences in users’ sentiments when comparing VR and AR use in higher education, as shown in Table 3. The results demonstrate that users’ sentiments, specifically in terms of positivity, anticipation, trust, and joy, were significantly higher for the use of VR in higher education compared to AR (p < 0.01). To further understand this variation, we examined the tweets associated with these sentiments. We found that anticipation, trust, and joy towards using VR were linked to its ability to inspire individuals to engage with interactive elements, allowing them to explore and navigate different learning stages. The tweets also highlighted VR’s potential to enhance users’ concentration on tasks, enabling them to think creatively and utilise available resources to solve complex problems through a trial-and-error approach. The results also indicated that AR was not perceived by users as inspiring them to spend more time understanding the sequence of learning, as it occurs in a non-iterative manner, thus distracting users and preventing sustained long-term engagement. Additionally, there was a lower sense of joy associated with AR compared to VR. Some users attributed this to AR’s tendency to present learning elements in a localised manner, which they might forget as the learning progresses.

**Table 3:** Descriptive statistics and ANOVA test results.

Sentiment	ARM (SD)	VRM (SD)	Sig
Anger	0.24 (0.06)	0.29 (0.03)	**–**
Anticipation	0.43 (0.09)	0.65 (0.06)	*p*** < **0.01
Disgust	0.12 (0.04)	0.19 (0.07)	**–**
Fear	0.34 (0.05)	0.31 (0.13)	**–**
Joy	0.34 (0.12)	0.49 (0.03)	*p = *0.00
Sadness	0.21 (0.07)	0.28 (0.06)	**–**
Surprise	0.18 (0.06)	0.32 (0.03)	*p* = 0.01
Trust	0.48 (0.13)	0.57 (0.08)	**–**
Negative	0.43 (0.08)	0.56 (0.05)	**–**

This bring us to assert that learners are likely to experience different emotions when using specific XR technologies. These findings add new knowledge to previous studies on individual engagement with and acceptance of VR and AR in different learning situations. For example, it extends the work of Fussell and Truong [43] by demonstrating how positive attitude towards using VR for dynamic learning can be shaped by users’ emotional association with the learning environment. This emotional connection not only can influence individuals’ engagement with VR-based learning activities but can also play a crucial role in shaping their behavioural intentions, motivation, and overall willingness to adopt VR as an effective learning tool. This line of thinking also aligns with the work of Calvert and Abadia [44], who reported positive emotions (e.g., interest, amusement, surprise, and elatedness) when using both VR and MR in a learning context.

## 5. Recommendations and practical implications

The comparison between VR and AR/MR use in learning offers various recommendations for higher education institutions in mapping their future strategies to optimise immersive capabilities for learning. For example, institutions might consider integrating VR into the current curriculum to enhance self-directed learning by providing immersive, interactive environments that support complex problem-solving. VR’s ability to promote autonomy and engagement makes it ideal for disciplines requiring visualisation, such as medicine, engineering, and the humanities. Meanwhile, investment in AI-driven adaptive VR environments can personalise learning experiences, facilitating deeper reflection and decision-making. VR has been found to stimulate creativity by enabling students to experiment with ideas in simulated environments through problem-solving scenarios and collaborative spaces. However, challenges such as motion sickness and cognitive load require strategic VR design adjustments, faculty training, and sustainable financial planning for effective implementation.

In addition, the use of AR in learning has the potential to provide guided and flexible learning through structured, hands-on experiences, particularly in medical and engineering fields. AI in AR applications can enhance engagement and comprehension through procedural learning tasks and progressive feedback. The results suggest that gamified elements should be integrated into AR curricula to support active learning, particularly in fields such as tourism, health sciences, and language studies. Despite its benefits, AR adoption poses challenges, including inconsistent integration into curricula, high costs, and interface usability concerns. This leads us to recommend that institutions develop standardised guidelines, invest in intuitive AR interfaces, and explore partnerships to ensure broader accessibility. Collaborative AR learning environments should also be enhanced through AI-driven interactions that adapt to students’ cognitive and emotional states.

On the other hand, this study offers a number of practical implications. First, the identification of topics in relation to VR and AR makes it easier for policy makers and technology practitioners to determine what type of technology is needed to support current teaching and learning practices. This is essential for those looking at redefining the current curriculum to incorporate immersive collaborative practices. Second, the identification of challenges and concerns regarding the use of XR technologies in higher education improves understanding for service providers on what measures to take in order to increase XR adoption by both university students and staff. Comparing the key challenges between VR/AR can guide digital technologists to seek workable solutions that can fit the needs of both students and academics. Third, identifying users’ sentiments can help designers of XR experiences customize the learning activity in a way that suits the emotional needs of learners, which can increase their long-term engagement and concentration. Fourth, the comparison between various emotional states in relation to the use of XR can also divert efforts to provide more responsive experiences that may otherwise be arbitrarily overlooked in a university education.

## 6. Limitations and future works

While the findings of this study provide valuable insights, it is important to address certain limitations that may affect the generalizability and interpretation of the results. The corpus used in this study consisted solely of English-language tweets, as English is one of the most widely used languages globally. This limitation resulted in the inclusion of 27,294 tweets, which may not fully capture a global perspective on XR use in the sector. This study was limited to the use of LDA as an example of an unsupervised learning algorithm to extract critical topics from the data. While this method has been widely employed in the literature for similar knowledge extraction tasks, future scholars could consider experimenting with alternative machine learning algorithms to uncover hidden themes in textual data. This includes comparing the results with other data collection methods such as questionnaire. Future research could also explore the use of different language models to capture and interpret users’ opinions about using XR technologies in learning, which was not possible in this study due to resource limitations. We also encourage future studies to attempt to identify associations between the topics identified in this study and users’ learning profiles through primary data collection methods, such as questionnaires or interviews. This approach could help researchers better understand how different educational levels approach the integration of XR in learning.

## 7. Conclusion

This study identified and compared key themes and sentiments related to the use of XR technologies in higher education. Our analysis revealed three primary themes for VR—self-directed learning, creativity-enhancing environments, and challenges and concerns—and three for AR/MR—guided and oriented learning experiences, playful and flexible learning experiences, and challenges and concerns. A key finding was the stronger emotional response associated with VR, particularly in terms of anticipation and trust, compared to AR/MR. These insights contribute to a better understanding of public perceptions of XR technologies in education, highlighting both opportunities and challenges. The study underscores the potential of VR and AR/MR to reshape learning experiences but also points to barriers that may hinder widespread adoption. Our findings provided important practical implications in the form of capturing and understanding key topics related to VR and AR use in learning. Higher education institutions can make informed decisions about the most suitable technologies to enhance teaching and learning. This is particularly valuable for those aiming to redesign curricula to incorporate immersive and collaborative practices. The identified challenges associated with XR adoption can pave the way for future developments and implementation of strategies that facilitate greater uptake among both students and faculty. The comparative understanding of VR and AR limitations allows digital technologists to develop targeted solutions that align with the needs of academic environments. Furthermore, examining the relationship between emotional states and XR use brings new knowledge on need for more responsive and adaptive learning environments that might otherwise be overlooked in higher education. These considerations offer as a foundation for future research and inform technology developers, educators, and policymakers in addressing current limitations and enhancing the effectiveness of XR in higher education.
